# Functional Aging in Male C57BL/6J Mice Across the Life-Span: A Systematic Behavioral Analysis of Motor, Emotional, and Memory Function to Define an Aging Phenotype

**DOI:** 10.3389/fnagi.2021.697621

**Published:** 2021-08-02

**Authors:** Shuichi Yanai, Shogo Endo

**Affiliations:** Aging Neuroscience Research Team, Tokyo Metropolitan Institute of Gerontology, Tokyo, Japan

**Keywords:** aging, animal models, inbred C57BL mice, behavior rating scale, spatial memory, locomotion, anxiety, handgrip strength

## Abstract

Aging is characterized generally by progressive and overall physiological decline of functions and is observed in all animals. A long line of evidence has established the laboratory mouse as the prime model of human aging. However, relatively little is known about the detailed behavioral and functional changes that occur across their lifespan, and how this maps onto the phenotype of human aging. To better understand age-related changes across the life-span, we characterized functional aging in male C57BL/6J mice of five different ages (3, 6, 12, 18, and 22 months of age) using a multi-domain behavioral test battery. Spatial memory and physical activities, including locomotor activity, gait velocity, and grip strength progressively declined with increasing age, although at different rates; anxiety-like behaviors increased with aging. Estimated age-related patterns showed that these functional alterations across ages are non-linear, and the patterns are unique for each behavioral trait. Physical function progressively declines, starting as early as 6 months of age in mice, while cognitive function begins to decline later, with considerable impairment present at 22 months of age. Importantly, functional aging of male C57BL/6J mouse starts at younger relative ages compared to when it starts in humans. Our study suggests that human-equivalent ages of mouse might be better determined on the basis of its functional capabilities.

## Introduction

The population of aged individuals continues to grow in many developed countries (Prince et al., 2015; WHO, 2015), and the human lifespan has dramatically increased around the world (Dong et al., 2016). While this can be seen as a benefit of modern technological, medical, and societal achievements, aging is still universal, unstoppable, and is still associated with progressive and overall physiological decline. Moreover, many older individuals become more and more impaired in their ability to generate adaptive functional responses and to maintain homeostasis under stress (Clegg et al., 2013). Consequently, the aged body becomes more susceptible to diseases, falls, delirium, disability, and injury, a condition called frailty (Fried et al., 2001; Vellas, 2016). As the term aging refers to the manifold changes that occur across an organism’s life-span (Kirkwood, 2005; da Costa et al., 2016), aging is thought of as occurring in an asynchronous and non-linear fashion in which several physical functions decline at the various rate (López-Otín et al., 2013; Rando and Wyss-Coray, 2021). The implications of these facts are that a holistic approach is needed in an aging research and that multiple physiological functions and behaviors of an aging organism need to be characterized at different stages of chronological age in order to properly describe an aging phenotype (Craig et al., 2015). Use of humans in aging research, however, is often problematic, because aging is modified by the complex interactions among accumulating biological alterations and environmental factors (Kirkwood, 2005; de Magalhães et al., 2012). And perhaps most importantly, the longer life-span of humans makes it difficult to study the biology of human aging over a reasonable time frame in research.

One of the guiding principles in aging research is that aging is universal and, as such, much can be learned about the biology of human aging by studying relevant model animals. Many feature the mouse model of aging, such as inbred mice (Yuan et al., 2009; Sundberg et al., 2011), hybrid mice (Miller and Nadon, 2000; Sumien et al., 2006), outbred and F1 mice (Chia et al., 2005), senescence-accelerated mouse (SAM; Takeda et al., 1981), and several lines of genetically modified mice (Kuro-o et al., 1997; Kõks et al., 2016). Even though hybrid line had some advantages over inbred lines (Yuan et al., 2009; Sundberg et al., 2011), the inbred C57BL/6J is the most commonly used animal model for aging research (Mitchell et al., 2015). Mouse strains with homogeneous genetic backgrounds are widely available; these mice are bred and housed in controlled laboratory environments to identify key processes involved in human aging and ultimately define an aging phenotype. The C57BL/6J mouse is characterized by its widespread availability and the role of background strain for the generation and breeding of genetically modified mice in the field of neuroscience (Ware et al., 2003; Crawley, 2008). Mice are also advantageous for aging research, because they have relatively short life-spans, more than 1/30th of that of humans (Goto, 2015; Mitchell et al., 2015; Dutta and Sengupta, 2016). A long line of evidence has established the laboratory mouse as the prime animal model for human aging studies (Kenyon, 2010; Mitchell et al., 2015). This large corpus of research has led to a better understanding of some of the neurochemical and behavioral changes associated with aging (Davis and Squire, 1984; Rosenzweig and Barnes, 2003; Bishop et al., 2010). Relatively little is known, however, about the details of behavioral and physical changes that occur across the life-span of model laboratory mice and the impact that aging has on behavior.

In the present study, we characterized functional aging in male C57BL/6J mice using a multi-domain behavioral test battery. The results derived from mice are translated to a human aging phenotype and discussed in terms of frailty (Fried et al., 2001; Vellas, 2016). This comparative knowledge offers a better way to understand disease mechanisms and senescence. This knowledge could inform the development of a preclinical mouse model of functional aging, a model that separates out the impact of aging on the phenotype from the impact of disease on the phenotype. Such a model has possible implications for clinical practice and public health.

## Materials and Methods

### Experimental Design, Power Analysis, and Statistical Analyses

We used a cross-sectional design with mice of different ages. A total of 211 mice were used; 117 of these mice were used for the battery of behavioral tests, and 94 mice were used for the water maze retention tests (below). A summary of the number of mice used is presented in Table 1. All mice used for the behavioral battery received all tests in the battery. To estimate the required sample size needed for the behavioral battery and the retention tests, we performed *a priori* power analysis using G^∗^Power software (Erdfelder et al., 1996; Faul et al., 2007). Our goal was to detect a relevant effect of aging with 95% actual power assuming that a one-way analysis of variance (ANOVA) was done using a significance level of *p* < 0.05. This analysis produced a required sample size of 125 mice for the behavioral battery and 125 for the retention tests. We originally planned using 120 mice for each experiment based on the power analysis. From the perspective of 3Rs (Russell and Burch, 1959), however, data on the Morris water maze task from the behavioral battery were also used for the analysis of the spatial memory retention experiment. This addition allowed us to achieve the required sample size to analyze the effect of age and retention of learned spatial location over short- and long-term intervals.

**TABLE 1:** Chronological ages and numbers of mice used.

Age cohort (months)	Group*	Number of mice included	Excluded (details)
3	Behavioral test battery	24	0
	Water maze retention	30	0
6	Behavioral test battery	23	1 (torticollis)
	Water maze retention	20	0
12	Behavioral test battery	24	0
	Water maze retention	18	2 (unknown)
18	Behavioral test battery	24	1 (cataract)
	Water maze retention	18	2 (cataract/unknown)
22	Behavioral test battery	22	2 (unknown)
	Water maze retention	18	2 (cataract/unknown)

*Mice in the water maze retention group were divided into two subgroups of retention intervals for each age cohort: one subgroup was tested for retention of platform location 10 days after the completion of acquisition training, and the other subgroup was tested for retention 30 days after acquisition training.

Our aim was to model a human aging phenotype in mouse that includes only a subset of deteriorative behavioral changes that reasonably captures the progressive deterioration of bodily functions over time. A wide range of behaviors were candidates that describe the condition of human aging (Rowe and Kahn, 1987; da Costa et al., 2016). Four behavioral traits associated with the peripheral nervous system and two traits associated with the central nervous system (CNS) were selected to represent a human aging phenotype (Table 2). The peripheral-derived traits (slowness, muscle weakness, reduced activity, and unintentional loss of body weight) were selected in reference to the clinical definition of the frailty syndrome (Fried et al., 2001; Vellas, 2016). For the CNS-derived traits, high-trait anxiety and cognitive impairment were used as part of the definition of a human aging phenotype, for which we selected corresponding mouse traits and operational measurements. The relevant literature sources for the comparative assessment adhered to meta-analyses of data collected from a range of young to elderly subjects by a national administrative organization (Table 2). However, articles on locomotor activity (Tveter et al., 2014) and cognition (Rossetti et al., 2011) report on studies that used population-based samples.

**TABLE 2:** Base set of traits for human aging phenotype, corresponding mouse traits, and operational measures for each trait.

Human aging phenotype	Mouse behavioral trait	Operational measure	References for human literature
-Unintentional weight loss	-Body weight	-Body weight*	Fryar et al., 2016 (Body weight)
-Reduced activity	-Locomotor activity	-Open field test -Home cage activity*	Tveter et al., 2014 (6-min walking test)
-Slowness	-Maximum gait velocity	-Rotor rod test*	Bohannon and Andrews, 2011 (Gait velocity)
-Weakness	-Grip strength	-Wire hanging test*	Bohannon et al., 2006 (Hand grip strength)
-Trait anxiety	-Anxiety	-Open field test* -Marble burying test	Tomitaka et al., 2015 (CES-D)
-Cognitive impairment	-Spatial memory	-Barnes maze -Morris water maze task*	Rossetti et al., 2011 (MoCA)
-Cognitive impairment	-Associative memory	-Pavlovian fear conditioning task	n/a

*Representative data for each behavioral trait of mouse is indicated by asterisk.

*CES-D, Center for Epidemiological Studies – Depression scale; MoCA, Montreal Cognitive Assessment; n/a, not available.*

Statistical differences among age cohorts were evaluated by one-way or two-way ANOVA with age cohorts as between-subject factor, as indicated in the text. Except for the power analysis, all analyses were performed using IBM SPSS statistics software for Windows, Version 23.0 (IBM, Tokyo, Japan). Statistical significance was set at *p* < 0.05. When the main effect was statistically significant, a Tukey–Kramer multiple comparison test was conducted to determine which means among the different age cohorts differed from the rest. All data are expressed as means ± S.E.M. Refer Supplementary Table for the summary of the statistical analyses.

### Subjects

Experimentally naive male C57BL/6J mice (CLEA Japan Inc., Tokyo, Japan) were used. Mice were housed in groups of four to five per cage [model# GM500; Tecniplast, Buguggiate (VA), Italy] with paper chips as bedding (PaperClean; Japan SLC, Inc., Hamamatsu, Japan). The mice were maintained in a specific pathogen-free (SPF) vivarium at 22 ± 1°C and 55 ± 5% humidity under a 12-h light-dark cycle (light on at 7:00 A.M.). Periodic microbial examinations were made to verify SPF throughout the entire housing period. Mice had free access to standard mouse feed (CRF-1, Oriental Yeast, Ltd., Tokyo, Japan) and chlorinated water (free chlorine, 2 ppm) throughout the entire experimental period. The mice health states were monitored daily by animal technicians. Mice were euthanized humanely once they show rapid and continuous weight loss or emaciation. All experiments were approved by the Animal Experiment Committee of the Tokyo Metropolitan Institute of Gerontology and were carried out according to its guidelines (Animal Protocol Approval Numbers 17012 and 20018).

### Apparatus

All apparatuses were obtained from O’Hara & Co., Ltd. (Tokyo, Japan), unless specified otherwise. We used Time^®^ automated monitoring software system (O’Hara & Co., Ltd.) to monitor the mice by video during behavioral testing in the open field test, Barnes maze, Morris water maze, and fear conditioning. Time^®^ was also used to monitor the home-cage activity of the mice online. The Time^®^ data acquisition system (O’Hara & Co., Ltd.) was used to control experimental devices and to analyze data.

### Behavioral Experiments

When the mice reached 3, 6, 12, 18, or 22 months of age, they were handled by the experimenters for 3 days, and then they were allocated to one of two groups: (1) the behavioral test battery group, or (2) the water maze retention group (Table 1). All behavioral experiments were conducted between 9 A.M. and 5 P.M.

### Procedures for Behavioral Test Battery

**FIGURE 1:**
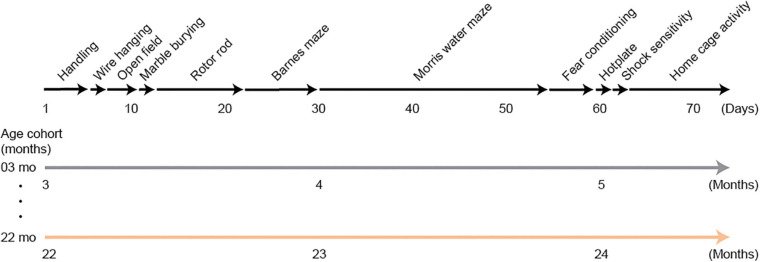
Schematic diagram of the experimental design for the behavioral test battery group. Mice were handled for 3 days, then they were subjected to a series of tests: wire hanging test, open field test, marble burying test, rotor rod test, Barnes maze, Morris water maze, Pavlovian fear conditioning task, hotplate test, electrical footshock sensitivity test, and home-cage activity assessment. Approximately 2 months were required to complete the whole test battery. Therefore, 3- and 22-month-old mice at the beginning of the experiment were about 5 and 24 months old, respectively, when they completed the experiment.

Mice in the behavioral test battery experiment were tested on the following tests: wire hanging test, open field test, marble burying test, rotor rod test, Barnes maze, Morris water maze, Pavlovian fear conditioning task, hotplate test, electrical footshock sensitivity test, and home-cage activity assessment. It took approximately 2 months to complete the whole test battery. A schematic diagram for this behavioral test battery is shown in Figure 1. Mice were transferred to each behavioral experiment room and habituated for 15 min before the beginning of each test. The order of the tests in the battery was administered so that the mice would be evaluated on less invasive tests before being administered tests that may consider to be more invasive (Crawley, 2008).

### Mouse Behavioral Traits and Operational Measures

#### Body Weight

Mice were weighed daily, and their weight recorded throughout the course of the behavioral experiment. Body weight measured at the beginning of the behavioral battery (i.e., first day of handling) was used in the statistical analysis of this trait.

#### Locomotor Activity

The behavioral trait we assigned for locomotor activity was extracted from data of mouse spontaneous activity measured in novel (open field test) and familiar (home cage) situations.

##### Open field test

Locomotor activity in a novel situation (Walsh and Cummins, 1976) was measured in an open field test, as we described previously (Kojima et al., 2008; Yanai et al., 2012, 2014). Briefly, a mouse was placed in the middle of the empty apparatus (50 cm × 50 cm walled box open on top) and allowed to explore this open field for 15 min on two successive days. Spontaneous behaviors were assessed under relatively dark illumination (10 lx) on the first day but under bright illumination (300 lx) on the second day. Throughout the 15 min, the mouse’s behavior was recorded via an overhead CCD camera, and the total distance traveled was determined by software. The number of rearings was also counted.

##### Home cage activity

Locomotor activity in a familiar situation was assessed by continuously monitoring the mice for spontaneous activity in their home cages. Mice were individually housed in cages containing wood-chip bedding. A 12-h light-dark cycle (light on at 7 A.M.) was used. After mice habituated to their new cage for 5 days, we recorded their behavior and calculated the total distance traveled on the sixth day (Yanai and Endo, 2016). Food and water consumption was continuously recorded for each mouse, and the total amount of food and water consumed on sixth day was calculated.

#### Gait Velocity

Gait velocity (Jones and Roberts, 1968) was calculated from mouse performance on the rotor rod task. We placed mice individually on a rotating rod according to a previously described protocol (Kojima et al., 2008). Once the mouse was on the rod, the rod’s axial rotational speed was increased progressively from 4 to 40 rotations per minute over a test period of 300 s. We recorded the total time the mouse remained on the rotating rod without falling. From this time and rotor rod speed, we then calculated velocity (cm/s), which was considered to be the mouse’s maximum gait velocity. Three trials were conducted per day for 5 consecutive days with each trial separated by a 15-min intertrial interval. The maximum gait velocity on day 5 was the value used for statistical analyses.

#### Grip Strength

The four-limb wire hanging test was used to assess grip strength and muscle endurance (Graber et al., 2013). Briefly, mice were forced to hang inverted onto a suspended stainless-steel wire grid (2 mm in diameter, wires spaced 1 cm apart) located 30 cm above the floor over a soft paper chip bedding (Kojima et al., 2008). After a mouse was placed at the center of a top lid with a wire grid, the lid was immediately closed so that the mouse was inverted inside of the apparatus. Latency to drop down from the wire grid was recorded (cutoff time, 300 s). Each mouse was tested twice with a 30-min intertrial interval. The longest latency of the two trials was used for analysis.

#### Anxiety-Like Behavior

Prevalence of emotional distress, including high-trait anxiety and depression, is a significant public health problem in the elderly (De Beurs et al., 1999; Richardson et al., 2011). Estimates of the prevalence of anxiety in old age varies widely from 1.2–14 to 1–28% in community versus clinical contexts, respectively (Remes et al., 2016). Prevalence is even higher in older people with mild cognitive impairment, ranging from 11 to 75% (Monastero et al., 2009; Yates et al., 2013). The corresponding behavioral trait of anxiety-like behavior in mice was behavior in the open field test and the marble burying test.

##### Open field test

Two behavioral indices were used to estimate anxiety-like behavior in a novel environment (Walsh and Cummins, 1976). For analysis, the open field was divided into virtual 25 squares; the central nine squares were defined as the center of the open field, and the 16 squares along the walls of the field were defined as the periphery. Over a test period of 15 min, total time spent in the periphery of the field and total immobility time, regardless of location, were measured for each mouse.

##### Marble burying test

Burying behavior of harmful or harmless objects by mice in their bedding material is a natural defense mechanism of mice that occurs under stressful conditions or states of anxiety (Njung’e and Handley, 1991). Our *a priori* hypothesis was that the number of marbles buried would be increasingly greater with increasing age, because marble burying is considered to reflect anxiety-like behavior (Broekkamp et al., 1986). For our assessment, mice were individually placed in their home cage containing 20 equally spaced glass marbles and were allowed to freely move around the cage for 5 min. At the end of 5 min, the number of marbles buried was counted and recorded. Some marbles were buried completely, but most often the marbles were partially buried. We counted a marble as being buried if two-thirds or more of it was covered (Wolmarans et al., 2016). We also measured each mouse’s total time being immobile during the 5-min test.

#### Memory

Numerous studies have documented an age-related decline in hippocampal-dependent declarative memory in humans (for review, Hoyer and Verhaeghen, 2006; Nyberg et al., 2012). For the purposes of the aging phenotype, we assessed hippocampus-dependent spatial memory using the Barnes maze (Barnes, 1979) and the Morris water maze task (Morris, 1981; Morris et al., 1982). Associative memory was assessed using a Pavlovian fear conditioning task (Fanselow, 1990; Phillips and LeDoux, 1992).

##### Barnes maze

The apparatus for this spatial memory task consists of elevated circular table (100 cm in diameter) with holes located around the periphery (Barnes, 1979; Gawel et al., 2019). The apparatus is placed in a brightly lit room with numerous visual cues on the walls. All of the 16 holes are open to the floor, except one hole that leads to a dark escape box on the underside of the platform. Since rodents naturally are motivated to avoid open spaces and bright lights, the mouse will try to find and crawl into the escape box as quickly as possible. Elapsed time and distance traveled to locate and enter the escape box are the main outcome measures; these are the escape latency and escape distance, respectively. As with the Morris water maze, rodents gradually learn the location of an escape location, reducing their time exploring. The mouse’s movements are monitored by an overhead camera, and distance traveled during each trial is also calculated.

During task acquisition training on the Barnes maze, the mouse was placed inside an opaque cylinder in the center of the apparatus for 10 s. At the end of this holding period, the cylinder was removed, and the mouse was allowed to freely explore the maze surface for a maximum of 180 s to find the fixed location of the hidden escape box. The mouse was left in the escape box for 60 s before being returned to its home cage. Three trials were conducted each day for 4 consecutive days, with each trial separated by a 30-min intertrial interval. One day after the completion of acquisition training, a 60-s probe test was done, in which all 16 holes were blocked. Time spent in the training quadrant and the number of times the mouse poked his nose into the hole where the escape box was located were taken as indices of spatial memory.

##### Morris water maze task

Spatial memory was also tested in the Morris water maze task (Morris, 1981; Morris et al., 1982), which shares some features of the Barnes maze. The water maze task was conducted as described previously (Yanai et al., 2014, 2018; Takahashi et al., 2019). During acquisition training, individual mice were allowed to swim freely for a maximum of 60 s in the pool (100 cm in diameter) to find the submerged escape platform. Four trials were conducted each day for 10 consecutive days, with an intertrial interval of 15 min. One day after the completion of acquisition training, a 60-s probe test was conducted with the escape platform removed from the pool. Because we observed significant age-related differences in swim speed (see Results) across age cohorts, the number of platform crossings in the probe test was standardized per 10 m of swimming distance.

On the day following the probe test, four consecutive days of cued training began. In the cued task, the location of the submerged platform was prominently marked by attaching a black flag to it.

##### Fear conditioning task

Mice were tested in a fear conditioning task to assess conditioned fear to both cue and context, as described previously (Kojima et al., 2008; Yanai et al., 2012; Takahashi et al., 2019). Briefly, mice were individually placed in a shocking chamber on the first day. After an exploratory period of 60 s, a conditioned stimulus (CS; 10 kHz, 70 dB pure tone) was presented for 3 s. The CS co-terminated with an unconditioned stimulus (US; 0.12 mA scrambled electrical footshock for 0.5 s). After the fear conditioning, mice were tested first for short-term (1 h) and then long-term (24 h) cue-dependent fear memory, followed by long-term contextual fear memory (48 h). For the cue-dependent fear memory tests, mice were placed individually into a new chamber to which they had never been previously exposed. This procedure separated out the contributions of cue-dependent fear memory from context-dependent fear memory; the tone was presented for 60 s. For the context-dependent fear memory test, mice were placed in the original shocking chamber, this time without receiving a footshock. Throughout the tests, duration of freezing behavior was measured and used an index of fear (Fanselow, 1990; Phillips and LeDoux, 1992). Based on impaired performance observed previously in the Barnes maze and the water maze task (Bach et al., 1999; Magnusson et al., 2003; Harburger et al., 2007; Weber et al., 2015), we hypothesized that aged mice would also be impaired in an associative memory task like fear conditioning.

#### Pain Sensitivity

After completion of the fear conditioning task, mice were tested on a separate day in the hotplate test, followed by the electrical footshock sensitivity test, as described previously (Kojima et al., 2008; Yanai and Endo, 2016; Yanai et al., 2018).

##### Hotplate test

In the hotplate test, pain sensitivity to heat was assessed by measuring the latency of the mouse to lick a forepaw after being placed on a 55°C hotplate (model MK-350HC, Muromachi Kikai, Tokyo, Japan).

##### Electrical footshock sensitivity test

The day after the hotplate test, sensitivity to electrical footshock was assessed by measuring the threshold electric current intensity to evoke a paw flick and vocalization. The mouse was placed in a shocking chamber, and a scrambled electrical footshock was progressively delivered, starting at an intensity of 10 μA and increasing by increments of 10 μA. The inter-stimulus interval was 15 s.

#### Short- and Long-Term Retention of Spatial Memory in Water Maze

A separate group of mice from those tested in the behavioral battery (Table 1) was used for assessing short- and long-term retention of spatial memory using the Morris water maze. Acquisition training was done exactly as the behavioral test battery. After this training, the mice were divided into two subgroups for each age cohort in such a way that achieved a balance in performance between the two subgroups in each age cohort. Then two different retention intervals were assessed for memory of the training platform location. For one subgroup, the retention interval was 10 days and the other was 30 days. One 60-s probe test was conducted at each of these retentions for each mouse. Consideration of the 3Rs (Russell and Burch, 1959) compelled us to include water-maze data from the behavioral test battery so that we would also have a 1-day retention interval, along with the two longer intervals tested in this experiment. These short-term retention data came from the behavioral test battery groups 1 day after the completion of acquisition training. Thus, we were able to examine the effect of age and retention interval at 1, 10, and 30 days after the acquisition training in mice aged 3, 6, 12, 18, and 22 months.

#### Scaling of Data

In order to examine the question of whether aging affects all behavioral traits equally, representative data from each behavioral trait were selected and scaled using the following formula:

Scaled performance = performance of each age cohort/maximum performance among all age cohorts.

Thus, maximum performance within a trait was assigned to 100. Based on plotted data, we determined age-related patterns for each behavioral trait by using a least squares method for each trait. Considering that divided attention during the encoding and recollection phase affects memory performance (Craik et al., 1996), we postulated that the precision of a spatial memory is a result of the amount of attention available for spatial information processing either at encoding and/or recollection. Consequently, we evaluated spatial memory separately in terms of high and low attention levels required.

## Results

In the present study, functional aging of male C57BL/6J mice were characterized using a multi-domain behavioral test battery. Prior to the beginning of the experiment, six mice were euthanized humanely due to rapid weight loss. In addition, one mouse was excluded due to torticollis and three mice were excluded due to cataract (Table 1). Other mice from each age cohort appeared normal at the beginning of behavioral testing. General health appeared good throughout all testing, and there were no signs of body postural abnormalities, tremors, convulsions, palpebral closure, exophthalmos, cataracts, or other abnormalities or behaviors (Irwin, 1968).

### Body Weight Increased Progressively With Increasing Age Cohort Despite Identical Energy Intake Across Cohorts

**FIGURE 2:**
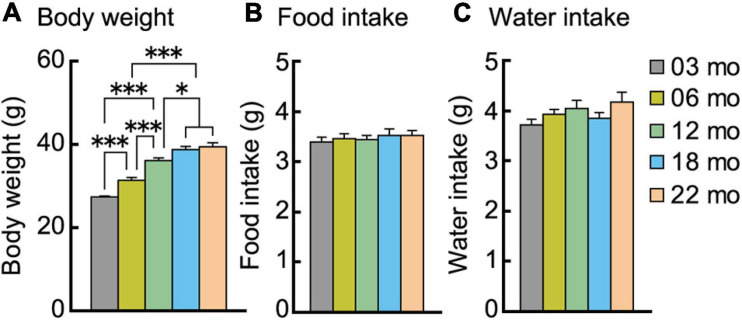
Body weight increases with increasing age despite similar food and water consumption. **(A)** Mean body weights measured at the beginning of the behavioral test battery for each age cohort are significantly greater with increasing ages. Mean daily individual consumption of **(B)** food and **(C)** water showed no significant effect of age. ^∗^*p* < 0.05, ^∗∗∗^*p* < 0.001. Error bars indicate ± SEM in this figure and subsequent figures.

We examined age-related differences in body weight and energy intake, i.e., food and water consumption. Body weights at the beginning of the behavioral test battery are shown in Figure 2A. There was a significant main effect of age on body weight (*F*[4,112] = 57.963, *p* < 0.001), with each progressively older age cohort significantly weighing more than the 3-month-old mice. However, the body weights of 18- and 22-month-old mice were statistically indistinguishable.

Food and water intake were continuously recorded in the home-cage environment. The average intake on the sixth day is shown in Figures 2B,C. No significant main effect of age was observed for food (Figure 2B) or water (Figure 2C) consumption. Thus, increasing age does not decrease or increase energy and fluid intake.

### Aged Mice Have Reduced Locomotor Activity Both in Novel and Familiar Environments

**FIGURE 3:**
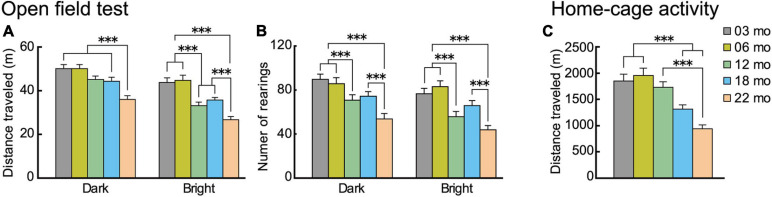
Locomotor activity decreases with increasing age in novel and familiar environments. Mean distance traveled and number of rearings of the different age cohorts in novel (open field) environment under low (dark) and high (bright) illumination and familiar (home cage) environment. In the open field test, **(A)** distance traveled and **(B)** number of rearings decreased significantly with increasing age and under both illumination conditions. **(C)** In the familiar environment of the home cage (sixth day), distance traveled decreased significantly with increasing age. ^∗∗∗^*p* < 0.001.

Locomotor activity of the different age cohorts was measured in novel environment under different levels of illumination (open field) and familiar environments (home cage) (Figure 3).

In the open field test, distance traveled and the number of rearings are two forms of locomotor activity in rodents (Walsh and Cummins, 1976). On thefirst day of testing, these behavioral indices were monitored under low illumination (dark condition), and on the second day, they were monitored under high illumination (bright condition). Regardless the illumination condition, locomotor activities of mice in all five age cohorts reached asymptote in 15 min (see Supplementary Figure 1). With increasing age, mice tended to travel less and less under both bright and dark conditions and in novel and familiar environments. For the distance traveled in the open field (Figure 3A), there was a significant main effect of age (*F*[4,112] = 14.348, *p* < 0.001) and interaction between age and illumination (*F*[4,112] = 3.924, *p* = 0.005). In the dark condition, 22-month-old mice traveled significantly shorter distances than the other four age cohorts, but in the bright condition, distance traveled varied among the different age cohorts and became significantly shorter with increasing age. Number of rearings also tended to decrease with increasing age (Figure 3B). There was a significant main effect of age (*F*[4,112] = 10.765, *p* < 0.001), with the number of rearings significantly decreasing with increasing age, both under dark and bright conditions. The performance of the two youngest age cohorts were virtually identical, with decreases in locomotor indices beginning to appear in 12-month-old mice in some conditions.

In home cage activity (familiar environment), the same general pattern of age-related decreased activity was also observed. Over the first 5 days of home-cage activity assessments (data not shown), home-cage activity of each age cohort progressively decreased, reaching asymptotic levels of distance traveled on the sixth day. For the distance traveled (Figure 3C), there was a significant main effect of age (*F*[4,112] = 14.418, *p* < 0.001), with older age cohorts traveling significantly shorter distances.

In summary, these results obtained from novel (open field) and familiar (home cage) environments show that locomotor activity in mice tend to decrease as a function of age under different environmental conditions.

### Gait Velocities Decrease With Increasing Age

**FIGURE 4:**
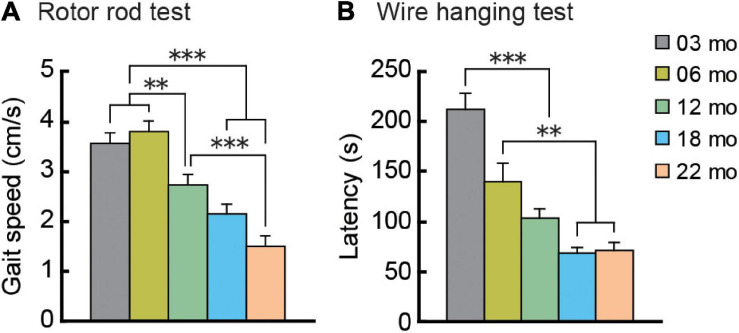
Gait velocity and grip strength decreases with increasing age. **(A)** Mean maximum gait velocity on the last training day (day 5) of the rotor rod test. **(B)** Grip strength as assessed by the wire hanging test. Mean latencies to fall from the wire grid are shown. Performance on these behavioral tests dramatically decreased with increasing age. ^∗∗^*p* < 0.01, ^∗∗∗^*p* < 0.001.

Mouse gait velocity was calculated from rotor rod performance. Maximum gait velocity of mice in all five age cohorts increased over 5 days of training as they gradually acquired the task under the conditions used, eventually reaching asymptotic levels (data not shown). Performance on the last training day was considered to be the maximum gait velocity (Figure 4A). Mean gait velocities decreased significantly with increasing age, as the main effect of age was significant (*F*[4,112] = 21.325, *p* < 0.001), and *post hoc* tests showed that the older cohorts’ gait velocities were significantly lower than those of two youngest cohorts.

### Grip Strength Decreases With Increasingly Older Age Cohorts

As an index of grip strength in mice, we calculated latency to drop from a wire grid in the wire hanging test. In general, mice in older age cohorts tended to fall from the hanging wire grid progressively sooner than mice in younger age cohorts, showing weaker grip strength (Figure 4B). One-way ANOVA revealed a significant main effect of age (*F*[4,112] = 21.104, *p* < 0.001), and subsequent *post hoc* tests showed that latency to fall got progressively shorter with increasing age cohort. In the 12-month-old and older cohorts, drop latencies were indistinguishable statistically.

### Anxiety-Like Behaviors Increase With Increasingly Older Age Cohorts

**FIGURE 5:**
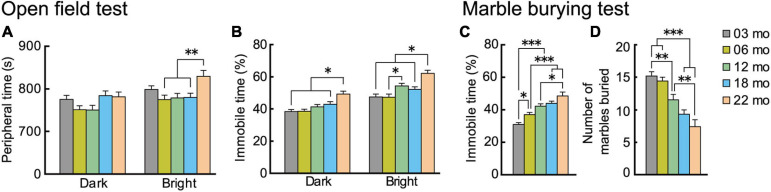
Trait anxiety increases with increasing age. Anxiety-like behaviors were evaluated with two types of tests: open field and marble burying. In the open field test, time spent in the periphery of the field was measured, and percentage of immobility time was calculated. Plotted are **(A)** mean time spent in the periphery, and **(B)** mean percentage of immobility time. In the marble burying test, percentage immobility time was measured, and total number of marbles buried was calculated. Plotted are **(C)** mean percentage of immobility time, and **(D)** mean total number marbles buried. Immobility times in both tests **(B,C)** generally increased significantly with increasing older age cohort. Contrary to expectations, number of marbles buried decreased with age. ^∗^*p* < 0.05, ^∗∗^*p* < 0.01, ^∗∗∗^*p* < 0.001.

Two types of tasks evaluated age-related changes in anxiety-like behavior. In the open field test, time spent in the periphery and time spent being immobile are indices of anxiety level (Walsh and Cummins, 1976). We tested the mice under dark and bright conditions. In general, with oldest age cohorts, mice spent more time in the peripheral section of the field, but this differed by illumination (Figure 5A). There was a significant main effect of age (*F*[4,112] = 3.460, *p* = 0.01), and the interaction between age and illumination was significant (*F*[4,112] = 2.988, *p* = 0.022). Twenty-two-month-old mice spent significantly more time in the periphery than mice in the other four age cohorts in the bright condition but not the dark condition. The age-related patterns were different for time spent being immobile in the open field test (Figure 5B). In general, immobility time was greater with increasing age cohort. The main effect of age was significant (*F*[4,112] = 10.893, *p* < 0.001), and there was a significant interaction between age and illumination (*F*[4,112] = 3.299, *p* = 0.014). The immobility time of 22-month-old mice was significantly greater than mice in the other four age cohorts under both dark and bright conditions. Thus, immobility time might be a more age-sensitive index to evaluate anxiety-like behavior in mice than time spent in the periphery of an open field.

Results of the marble burying test generally corroborated our observations of increased anxiety-like behaviors with increasing age in the open field. As with the age-dependent increase in immobility time in the open field test, in the marble burying test, immobility time also increased with increasingly older age cohorts (Figure 5C). The main effect of age was significant (*F*[4,112] = 18.502, *p* < 0.001), and time spent being immobile got significantly longer with each increasing age cohort. We observed a different type of outcome for the marble burying index of anxiety-like behavior. While our *a priori* hypothesis was that the number of marbles buried would increase with increasing age, reflecting increased anxiety (Broekkamp et al., 1986), we instead observed that the number of marbles buried decreased significantly with increasing age cohort, as indicated by the main effect of age (Figure 5D; *F*[4,112] = 17.809, *p* < 0.001). Although the marble burying test is widely used as an index for anxiety, there is also controversy over the interpretation and validity of its results (Borsini et al., 2002; Wolmarans et al., 2016). Previous studies reported that the marble burying may be more related to repetitive digging behavior (Thomas et al., 2009) or obsessive-compulsive disorder-like behavior (Taylor et al., 2017). It should be noted, however, that the age-dependent increase in immobility time was observed in the marble burying task (Figure 5C).

In summary, the results suggest that anxiety-like behaviors increase with increasing age. This was especially apparent in 22-month-old mice, as ected in immobility time in both the open field and marble burying tests.

### Acquisition and Retention of Spatial Memory Are Impaired in the Oldest Mice

Considering the age-related decline in hippocampal-dependent memory documented in humans (Hoyer and Verhaeghen, 2006; Nyberg et al., 2012) and other species (Barnes, 1979; Rapp et al., 1997; Rosenzweig and Barnes, 2003; Coppola et al., 2014), we examined hippocampus-dependent learning and memory function in the different age cohorts of mice in two different spatial memory tasks.

#### Barnes Maze

**FIGURE 6:**
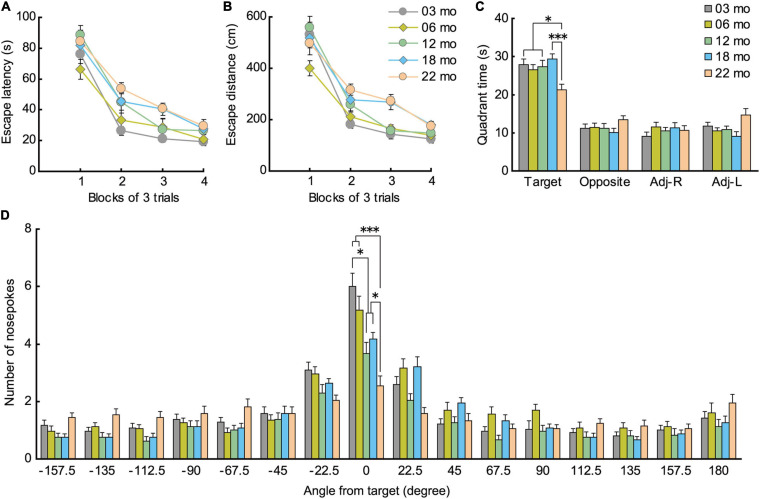
Impaired spatial memory in the Barnes maze. **(A)** Mean escape latency (s) and **(B)** mean escape distance (cm) to find the hidden escape box during 4 days of consecutive training (3 trials per day). Latencies and distance traveled were significantly longer for 22-month-old mice compared to 3-month-old mice. **(C)** In the probe test, 22-month-old mice spent significantly less time in the quadrant where the escape box was located previously. **(D)** Number of nose pokes in the target hole also indicated that memory for location of the training escape hole was impaired in 12-month-old mice and all older age cohorts compared to 3-month-old mice. ^∗^*p* < 0.05, ^∗∗∗^*p* < 0.001.

During acquisition training in the Barnes maze, mice gradually learned to locate the hidden escape box, as indicated by a progressive reduction in escape latency (Figure 6A; *F*[3,336] = 219.725, *p* < 0.001) and escape distance (Figure 6B; *F*[3,336] = 180.888, *p* < 0.001). There was also a significant main effect of age in escape latency (*F*[4,112] = 3.252, *p* = 0.015), with significantly longer escape latencies in 22-month-old mice compared to 3-month-old mice. We also observed a significant main effect of age for escape distance (*F*[4,112] = 6.403, *p* < 0.001). *Post hoc* tests showed that escape distances of 18- and 22-month-old mice were significantly longer than that of 3- and 6-month-old mice. Since older mice tend to have lower levels of locomotor activity (Figures 3A–C) and display greater anxiety-like behaviors (Figures 5A–C), escape distance, rather than escape latency, might better reflect age-related changes in learning ability in the Barnes maze.

In the probe test 1 day later, 22-month-old mice spent significantly less time in the quadrant of the platform where the escape hole was (Figure 6C), as indicated by the significant main effect of age (*F*[4,112] = 4.458, *p* = 0.002) and subsequent *post hoc* comparisons of the time spent in the training quadrant. Older age cohorts also had significantly fewer nose pokes in the training hole, as indicated by a significant main effect of age (Figure 6D; *F*[4,112] = 11.092, *p* < 0.001), and *post hoc* tests showed that this impairment appeared first in 12-month-old mice and progressively worsened with age. These results show that older mice have poorer spatial memory for the training quadrant and specific escape hole that they acquired over the previous 4 days of spatial training, a deficit that may begin to appear as early as 12 months of age in mice.

#### Morris Water Maze Task

**FIGURE 7:**
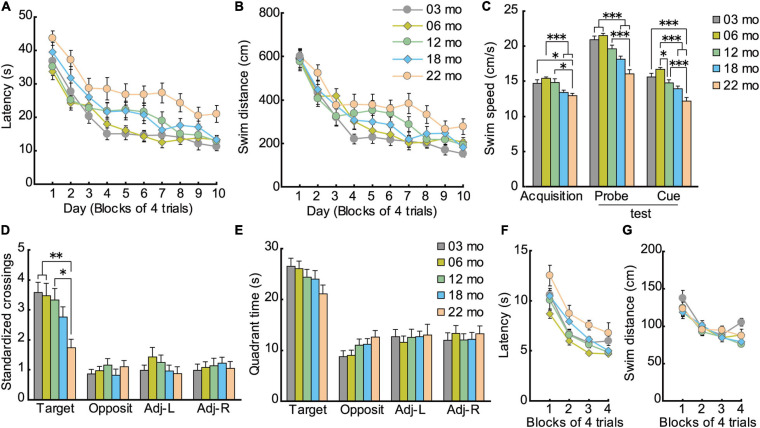
Impaired spatial memory in the Morris water maze. **(A)** Mean escape latencies and **(B)** mean swim distances to the submerged platform over 10 days of training. Performance of each cohort gradually improved over 10 days, eventually becoming asymptotic by day 10. **(C)** Swim speeds of older mice were significantly slower and swim distances significantly longer, suggesting that the appropriate index to evaluate spatial learning ability is swim distance. **(D)** Mean number of platform crossings during the probe test, which was carried out 1 day after the completion of training. **(E)** Mean time spent in the training quadrant during the probe test. In 22-month-old mice, platform crossings were significantly fewer than in younger mice, suggesting that 22-month-old mice were significantly impaired. **(F)** Mean escape latencies and **(G)** mean swim distances to the visible platform during cued training. ^∗^*p* < 0.05, ^∗∗^*p* < 0.01, ^∗∗∗^*p* < 0.001.

Similar to acquisition performance in the Barnes maze, in the Morris water maze task, the mice also gradually learned to locate and swim to the hidden escape platform. Both the mean escape latency (Figure 7A; *F*[9,1008] = 68.959, *p* < 0.001) and swim distance (Figure 7B; *F*[9,1008] = 71.682, *p* < 0.001) decreased significantly over 10 days of training. Visual inspection of the graphed results appear to show that 22-month-old mice performed relatively poor in spatial memory acquisition. The main effect of age for both escape latency (*F*[4,112] = 8.614, *p* < 0.001) and swim distance (*F*[4,112] = 4.582, *p* = 0.002) were significant. *Post hoc* comparisons revealed that 22-month-old mice were significantly impaired on both of these measures compared to 3-month-old mice. Swim speed during acquisition training was also age-dependent, as indicated by a significant main effect of age (Figure 7C; *F*[4,112] = 6.814, *p* < 0.001) and *post hoc* tests. Considering that swim speeds become slower in an age-dependent manner, the use of swimming distance to evaluate spatial learning ability is appropriate (Yanai and Endo, 2016; Yanai et al., 2018).

Given that swim speeds differed significantly by age during task acquisition (Figure 7C; *F*[4,112] = 19.514, *p* < 0.001), number of crossings over the previous location of the escape platform was standardized per 10-m swim distance in the probe test. Thus, the main effect of age for number of crossings was significant (Figure 7D; *F*[4,112] = 4.329, *p* = 0.003), even though the five age cohorts had spent similar time in the training quadrant statistically (Figure 7E). *Post hoc* tests showed that 22-month-old mice made significantly fewer platform crossings compared to three younger cohorts of mice.

The disparity in results between the time spent in the target quadrant and the number of platform crossings may be related to differences in the relative degree of attention required for performance. The idea that age-related attention deficits might explain some aspects of memory deficits in rodents finds its roots in the classic studies of Craik et al. (1996) on divided attention on encoding and retrieval processes in human memory. In our case, the encoding of the general spatial location (trained quadrant) may be augmented by further elaborate processing (corresponding roughly to the amount of attention paid to spatial cues) to learn the precise platform location. Such further processing requires additional processing resources that may not be as readily available with increasing age. Therefore, with aging, perhaps less attention can be allocated to spatial information processing required for precise location (Figure 7D), but there is sufficient attention allocated for previously learned quadrant (Figure 7E). Altogether, performance in the probe test in the Morris water maze can be classified into two types of memory dependent on level of attention required: general (quadrant) and precise spatial location (platform). The two measures used to assess a previously learned spatial location in the water maze, then, are more or less sensitive in detecting age-related differences in spatial memory (Maei et al., 2009; Yanai et al., 2014).

As with acquisition in the hidden platform test, mice also gradually learned to locate the visible platform in cued training of the Morris water maze task. The main effect of training for escape latency was significant (*F*[3,336] = 45.203, *p* < 0.001), and there was a significant effect of age (Figure 7F; *F*[4,112) = 4.934, *p* < 0.001). *Post hoc* tests showed that 22-month-old mice had significantly longer escape latencies than 6- and 12-month-old mice. Because older mice swam significantly slower than younger mice (Figure 7C; *F*[4,112] = 19.514, *p* < 0.001), their longer escape latencies may be due in part to their reduced swim velocity. No significant differences in swim distance were found among the five cohorts of mice (Figure 7G; *F*[4,112] = 1.768, *p* = 0.140). This result suggests that visual abilities among the different age cohorts were sufficiently good to discriminate spatial stimuli needed to locate and navigate to a visible escape platform.

The effect of aging on spatial memory has been extensively studied using the Barnes maze (Barnes, 1979; Gawel et al., 2019) and the Morris water maze task (Morris, 1981; Morris et al., 1982) in rodents (for review, Kennard and Woodruff-Pak, 2011). Both tasks depend on a sufficiently functioning hippocampus that allows animals to learn the relationship between a fixed escape location and distal cues in the surrounding environment. One of the criticisms of the Barnes maze is that it is less sensitive to detecting behavioral changes of genetically modified mice than is the Morris water maze task (Stewart et al., 2011). In the present study, however, spatial memory impairment was detected as early as 12 months of age in the Barnes maze probe test (Figure 6D), as previously reported (Bach et al., 1999; Weber et al., 2015). In contrast, impairment of the water maze was detected only in the 22-month-old mice (Figure 7D). This difference in these similar spatial tasks may be due to differences in stress levels related to motivation to solve the task: the Barnes maze uses bright lights; the water maze uses water escape. In rodents, being immersed in water can lead to high corticosterone levels, which may affect performance of the mice (Holmes et al., 2002; Harrison et al., 2009). Thus, the Barnes maze may be less stressful for mice than the Morris water maze task. We decided to train the mice in memory tasks; 4 days for Barnes maze and 10 days for Morris maze based on the acquisition of the target quadrant is clearly established. Thus, we are able to compare all the age cohorts under the same condition. The other way will be to continue training the mice until no significant difference in target acquisition (Lione et al., 1999). Further research using latter training in addition to current training is necessary for the precise evaluation of mice memory in aging.

### Aged Mice Showed Enhanced Freezing in Pavlovian Fear Conditioning

**FIGURE 8:**
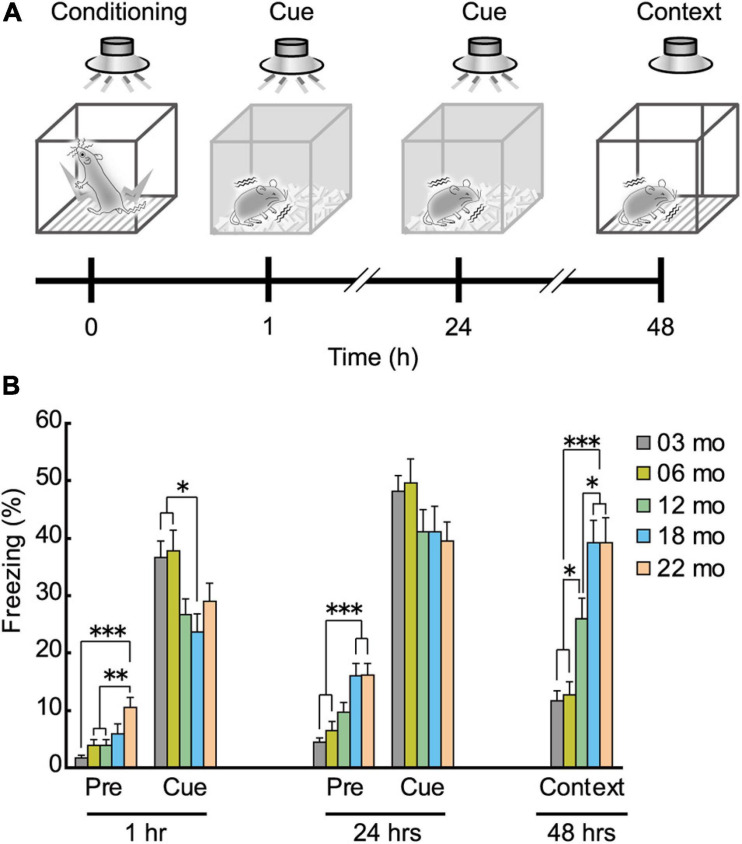
Enhanced freezing of aged mice in Pavlovian fear conditioning. **(A)** Schematic diagram of the fear conditioning task, illustrating the elements of training, short-term and long-term memories for the cue, and long-term contextual memory. After conditioning with the tone (CS) and electrical footshock (US), conditioned freezing was tested at 1 and 24 h after the conditioning (cue-dependent fear memory). Context-dependent fear memory was then tested 48 h after the conditioning. **(B)** Mean percentage of conditioned freezing in the fear conditioning task. Age-dependent increase in conditioned freezing was observed before tone presentation (pre-tone phase) in short- and long-term cue-dependent fear memory test, and context-dependent fear memory test. ^∗^*p* < 0.05, ^∗∗^*p* < 0.01, ^∗∗∗^*p* < 0.001.

As shown in schematic diagram in Figure 8A, mice were conditioned with a paired presentation of tone (CS) and footshock (US), and sequentially tested for short-term (1 h after conditioning) and long-term (24 h after the conditioning) cue-dependent fear memory. Long-term contextual fear memory was tested 48 h after the conditioning. Based on the impaired performance observed in the Barnes maze (Figure 6) and the Morris water maze task (Figure 7), we hypothesized that aged mice would also show impairment in the fear conditioning task. In the short-term cue-dependent fear memory test, the main effect of age was significant (*F*[4,112] = 3.905, *p* = 0.005). Subsequent *post hoc* tests showed that percentage of conditioned freezing in 3- and 6-month-old mice was significantly greater than in 18-month-old mice (Figure 8B). Although older mice tended to show numerically less conditioned freezing in the long-term cue-dependent fear memory test, statistically, average freezing behavior of the five age cohorts was statistically indistinguishable (*F*[4,112] = 1.514, *p* = 0.203, n.s.). For long-term context-dependent fear memory, percentage of conditioned freezing was significantly greater with increasing age cohort (*F*[4,112] = 16.503, *p* < 0.001).

The percentage of freezing behavior before tone presentation was also age-dependent (Figure 8B). In 22-month-old mice, significantly more freezing was observed than in the three younger cohorts of mice during the pre-tone phase in the short-term cue-dependent fear memory test (Figure 8B, 1 h; *F*[4,112] = 6.967, *p* < 0.001). During the pre-tone phase of the long-term cue-dependent fear memory test (Figure 8B, 24 h), 18- and 22-month-old mice exhibited significantly more freezing than 3- and 6-month-old mice (*F*[4,112) = 10.005, *p* < 0.001).

**FIGURE 9:**
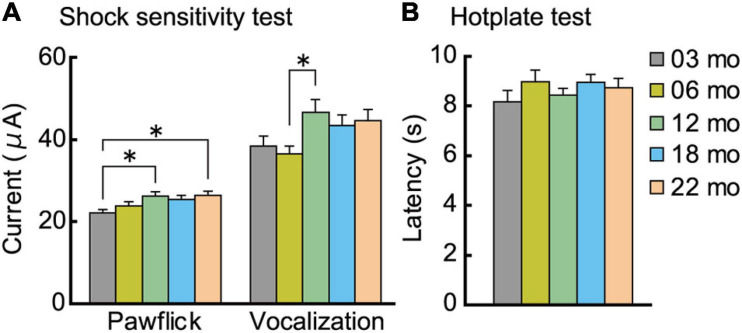
Analgesia tests. Pain sensitivity to electrical and thermal stimuli was assessed. **(A)** A subtle but significant age-related increase was observed for paw flick and vocalization to electrical footshock. **(B)** In the hotplate test, sensitivity to thermal stimuli was indistinguishable across the different age cohorts. ^∗^*p* < 0.05.

After the completion of the fear conditioning task, we examined the possibility that the enhanced conditioned freezing of older mice in the contextual fear memory test (Figure 8B) may be due to increased sensitivity to the noxious electrical footshock. A subtle but significant age-related increase was observed for paw flick (*F*[4,112] = 3.309, *p* = 0.013) and vocalization (*F*[4,112] = 2.820, *p* = 0.028) threshold (Figure 9A). Thus, blunted sensitivity to electrical footshock may not account for the enhanced conditioned freezing of older mice for contextual fear memory. However, it should be noted that pain sensitivity to thermal stimuli appeared to be similar among the different age cohorts (Figure 9B).

Another possibility is that intense freezing observed in older aged mice may reflect elevated anxiety states (Figures 5A–C) and/or reduced locomotor activity (Figures 3A–C). Previously, we had optimized the fear conditioning protocol for young mice (Yanai et al., 2014), and this protocol led to the saturation of freezing behavior in aged mice (i.e., ceiling effect). An alternative explanation for the enhanced freezing in aged mice might be related to impaired performance in behavioral pattern separation in mice (Creer et al., 2010; Cès et al., 2018), which also occurs in humans (Yassa et al., 2011; Stark et al., 2013). Pattern separation is the process by which subtle changes between successive tests are detected. More detailed research is required to disentangle the contributions of aging *per se*, emotional states, pain sensitivity, and/or other cognitive factors to that can influence fear memory.

### Short- and Long-Term Retention of a Learned Spatial Location Appears to Decay More Quickly in Aged Mice

**FIGURE 10:**
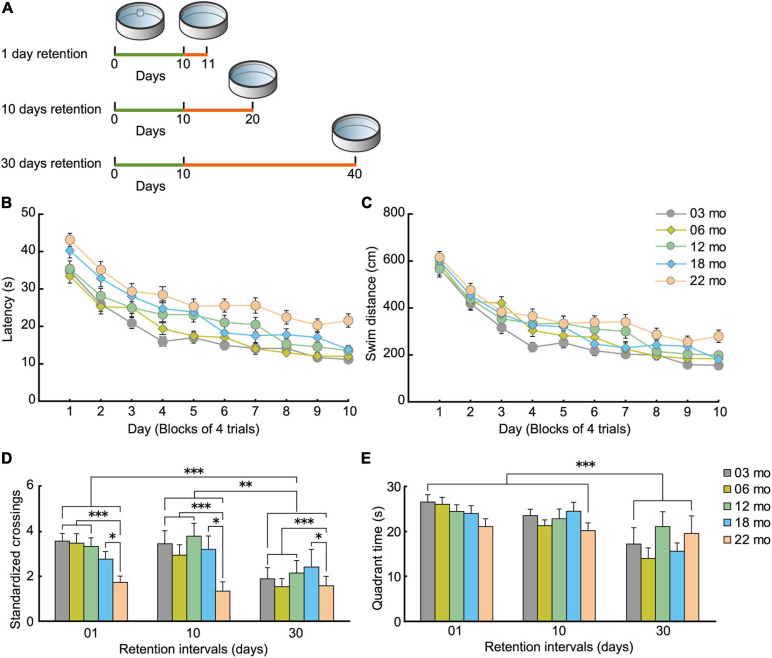
Decay of acquired spatial memory in the Morris water maze. **(A)** Experimental scheme for how retention interval affects the Morris water maze probe test. For each age cohorts (3, 6, 12, 18, and 22 months), three different interval subgroups were arranged. After 10 days of spatial acquisition training, a 60-s probe test was conducted after 1-, 10-, or 30-day intervals. Green line indicates the spatial acquisition training, and orange line indicates the retention interval. Retention data on day 1 were obtained from the behavioral test battery experiment. **(B)** Mean escape latency and **(C)** mean swim distance to the submerged platform during spatial acquisition training. **(D)** Mean number of platform crossings during the probe test, and **(E)** mean time spent in the trained quadrant during the probe test. Acquired spatial memory was retained for at least 10 days, but decayed at 10 and 30 days. This forgetting process was nearly equivalent in all five age cohorts. ^∗^*p* < 0.05, ^∗∗^*p* < 0.01, ^∗∗∗^*p* < 0.001.

In the formation of memory, the process of forgetting is equally important as the process of memory acquisition and retention (Hardt et al., 2013; Sadeh et al., 2014). We also assessed longer retention intervals of a previously learned spatial location in the different age cohorts to further build up a behavioral phenotype of functional aging in mice. A schematic diagram for this retention experiment is shown in Figure 10A.

As with the water-maze results from the behavioral battery (Figures 7A,B), mice in the spatial memory retention task gradually learned the location of the submerged escape platform in the water maze. The main effects of age on escape latency (Figure 10B; *F*[4,205] = 15.431, *p* < 0.001) and swim distance (Figure 10C; *F*[4,205] = 6.743, *p* < 0.001) were significant during task acquisition, and *post hoc* tests revealed that 22-month-old mice had significantly longer escape latencies and swim distances. For the probe test of the learned spatial location of the platform (Figure 10D), 22-month-old mice had the worst performance numerically at 1-, 10-, and 30-day retention intervals. In general, performance of the other younger age cohorts was similar for the 1- and 10-day retention intervals but was much worse at the 30-day retention interval. The main effect of age on the number of standardized platform crossings (*F*[4,195] = 4.324, *p* = 0.002) was significant in the 60-s probe test, and *post hoc* tests showed that performance of 22-month-old mice was significantly worse than the other age cohorts of mice (Figure 10D). In terms of the time spent in the training quadrant during the probe test, the five age cohorts spent a similar amount of time regardless of retention intervals (Figure 10E). We observed a significant main effect of retention interval on the number of platform crossings (Figure 10D; *F*[2,195] = 6.895, *p* < 0.001) and time spent in the training quadrant (Figure 10E; *F*[2,195] = 13.188, *p* < 0.001). *Post hoc* comparisons revealed that platform crossings at the 30-day-retention interval were significantly lower than at the 1- and 10-day-retention intervals. This result suggests that a previously acquired spatial memory is retained at least for 10 days and decays somewhat between 10 and 30 days, or becomes less accessible at those longer retention intervals. Since there was no significant interaction between age and retention interval for either number of platform crossings or quadrant time, one conclusion is that the decay of acquired spatial memory is working similarly among the five age cohorts. Alternatively, the performance indices we used to measure memory retention may be insufficiently sensitive to detect differences.

### Aging Does Not Affect All Behavioral Traits Equally in Mice

**FIGURE 11:**
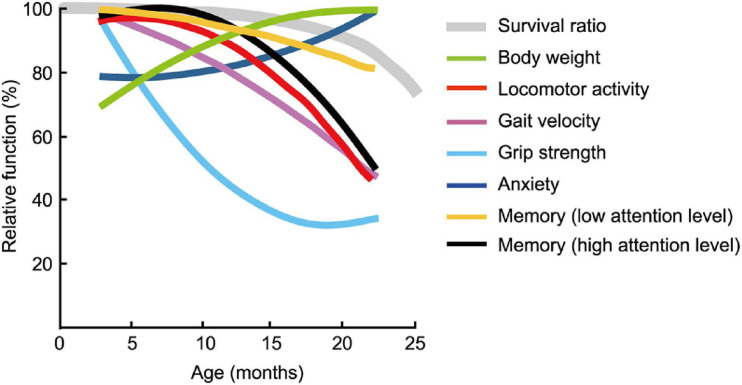
Pattern of functional aging differs depending on behavioral trait. Estimated age-related patterns of representative performance for each trait plotted as a function of age. Least squares method was used to determine age-related patterns from representative data of behavioral test battery (Table 2). With the exception of body weight and trait anxiety, behavioral traits across age cohorts had different rates and patterns of decline. Survival rate of the mouse is adapted and modified from Goto (2015).

The estimated age-related patterns for the seven behavioral traits changed differently with increasing age; some were markedly different (Figure 11) and were definitely not monotonically declining. Physical functions started to decline at 6 months of age for grip strength (Figure 4B) and at 12 months of age for locomotor activity (Figures 3A,C) and maximum gait velocity (Figure 4A). Others have also reported declines in wire-hang latency, rotor rod performance, open field locomotor activity as early as 4–7 months of age in male C57BL/6J mice (Shoji et al., 2016). Among tests measuring behavioral traits associated with the peripheral nervous system, grip strength has the most sharper rate of decline with increasing age compared to gait velocity and locomotor activity. As hand grip strength in middle-age predicts functional limitations and disability in human aging (Rantanen et al., 1999), grip strength in mice may be a useful measure for predicting deterioration of overall physical function. Including this trait in an aging mouse phenotype is consistent with the idea of a testing “toolbox” for assessing “healthspan” in aging mice (Bellantuono et al., 2020).

A multi-domain behavioral test battery used in this study is distinct from “toolbox” (Bellantuono et al., 2020) in term of describing the condition of human aging associated with the CNS, in addition to the peripheral nervous systems. Considering that the rate at which performance in some cognitive tasks declines across the life span may depend on the required level of attention to learn and remember (Craik et al., 1996), we postulated that the precision of a spatial memory is a result of the amount of attention available for spatial information processing either at encoding and/or recollection. Consequently, we evaluated spatial memory separately in terms of high and low attention levels required (see also results of the Morris water maze). We found that the rate at which performance of some cognitive tasks declines with increasing age depends on the required level of attention to learn and remember (Craik et al., 2010). For example, memory performance that requires a high level of attention—like remembering the precise location of the platform in the Morris water maze (Figure 10D) —has a sharper rate of decline with increasing age than memory requiring lower levels of attention—like remembering the trained quadrant where the escape platform was located (Figure 10E). We speculate that with increasing age, mice have less attention to allocate to spatial memory encoding, explaining why their performance is worse in some aspects of spatial memory (e.g., platform crossings) while seeming apparently normal on other aspects (e.g., time spent in the quadrant). Generally, old mice are considered to exhibit cognitive impairment. However, our results emphasize that multiple parameters of memory performance must be considered when defining an aging phenotype.

## Discussion

Aging is a complex process (Kirkwood, 2005; de Magalhães et al., 2012) and has been compellingly argued recently that aging may occur in an asynchronous, non-linear fashion across cells and tissues and space and time (López-Otín et al., 2013; Rando and Wyss-Coray, 2021). If cellular and tissue aging is not uniform across time or space, how might this be manifested in the aging mouse? Unfortunately, relatively little is known about how behavior, cognition, and physical functions in mice change across the lifespan. In the present study, we tested 3-, 6-, 12-, 18-, and 22-month-old male C57BL/6J mice in order to characterize functional aging across the life span (Figures 2–10).

Our results yielded three main insights about a mouse model of aging from a holistic perspective and how they relate to aging in humans. First, physical function as assessed by the wire hanging test (grip strength) progressively declines starting as early as 6 months of age in male mice, while cognitive function that requires a low level of attention begins to decline later in life, with observable impairment appears at 22 months of age. Second, functional aging of the C57BL/6J male mouse starts at younger relative ages compared to when it starts in humans. Finally, mice between the ages of 18 and 24 months are generally considered “old” (Flurkey et al., 2007), however, aging of mouse should be judged on the basis of its physiological functions. This outcome raises the question about what behavioral traits and physical functions in mice best describe the aging phenotype and whether these models are adequate for the phenotype of aging in humans (Bellantuono et al., 2020).

### Functional Aging in Mice and Humans

Humans and mice have very different lifespans but show similar patterns in disease pathogenesis and organ and systemic physiology (Demetrius, 2006). Thus, the C57BL/6J mouse strain has been a useful animal model to define an aging phenotype (Kenyon, 2010; Mitchell et al., 2015). A fundamental question arises here: How old are mice in human years? Using corresponding functional age—as assessed with our multidomain behavioral test battery—rather than chronological age, may be more appropriate for a mouse model of human aging.

**FIGURE 12:**
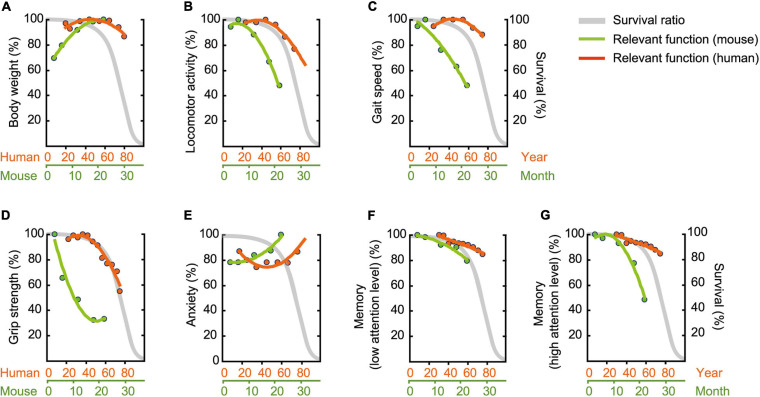
Assessment of functional aging in humans and mice. Age-related patterns were determined based on representative data (Table 2), and then superimposed onto survival rate. **(A)** Body weight, **(B)** locomotor activity, **(C)** gait velocity, **(D)** grip strength, **(E)** trait anxiety, **(F)** memory requiring low attention level, and **(G)** memory requiring high attention level. Survival rate is adapted and modified from Goto (2015). Original drawing is provided here courtesy of Goto (2015).

To compare age-related behavioral alterations of male mouse (Figure 11) and human, we also scaled age-related patterns for human behavioral phenotype from representative data (Table 2) employing the same methods we used to determine age-related patterns for mouse behavioral trait. When one compares the physiological function of different species (e.g., human versus mice), known criteria common to both species, such as survival rate, are indispensable. Although the maximum life-span of humans and mice differ, the shapes of the survival rate curves are remarkably consistent (Mitchell et al., 2015). On the basis of this facts, we used survival curve as a calibration reference for chronological age (Figures 12A–G).

### Body Weight Gain in Mice Over the Life Span

Over a human lifetime, body weight increases with age in a gradual parabolic manner, typically reaching a maximum in the fifth decade of life (Figure 12A; Fryar et al., 2016), and then tends to decline afterward. Surprisingly, we did not observe this later-in-life body weight loss in our male C57BL/6J mice (Figure 2). Rather, body weight rapidly increased up to 18 months of age, apparently reaching asymptote between 18 and 22 months of age (Figure 12A). This species difference in age-related body weight over the life span may be due to differences in the individual’s balance between daily energy intake and expenditure, as weight loss in elderly people (Zhu et al., 2010; Giezenaar et al., 2016) is due to reduced daily energy intake relative to energy expenditure (Speakman and Westerterp, 2010). In contrast, mice in the present study maintained a constant energy intake (Figures 2B,C) and locomotor activity decreases as a function of age (Figures 3A–C). Considering the critique that *ad libitum* fed animals frequently leads to overeating and excessive gain of body weight (Sohal and Forster, 2014), it is problematic to identify corresponding human age based on mouse body weight. Further investigation on the behaviors using calorie-restricted mice may overcome the issues (Kuhla et al., 2013; Ma et al., 2018).

### Physical Aging of Mice Is More Accelerated Than We Had Expected

Compared to humans, we found declines in locomotor activity (Figure 12B), gait velocity (Figure 12C), and grip strength (Figure 12D); these declined at younger relative ages in male mice compared to humans. Age-related changes in grip strength assessed by the four-limb wire hanging test is controversial (DeanIII, Scozzafava et al., 1981; Graber et al., 2013; Shoji et al., 2016; Börsch et al., 2021), however, several studies have demonstrated that these physical traits apparently deteriorate in aged mice (DeanIII, Scozzafava et al., 1981; Benice et al., 2006; Serradj and Jamon, 2007; Graber et al., 2013). DeanIII, Scozzafava et al. (1981) showed, for example, that performance in the wire-hang test begins to decline between 23 and 31 months of age. By contrast, our mice began to show some signs of decreased physical function as early as 6 and 12 months, respectively, for grip strength (Figure 4B) and locomotor activity (Figures 3A–C, 8A). In humans, peak athletic performance that requires strength and speed has been well-documented to occur before mid-life (Guillaume et al., 2011), and over the life span, takes the form of an asymmetrical inverted-U shape with a slower decline in later years (Berthelot et al., 2019). Our results appear to show that this kind of physical function peaks relatively earlier in male C57BL/6J mice compared to humans and begins to decline sooner and at a faster rate (Figures 12B–D). Based on locomotor activity (Figure 12B) and gait velocity (Figure 12C), it might be reasonable to consider male mice that are 12 months of age correspond roughly to elderly humans more than 60 years old. However, rigorous argument might be required when comparing the gait of bipedal human and sedentary mice. Together with possible contribution of body weight (Oellrich et al., 2016) and muscle mass (Börsch et al., 2021), further careful studies using other tests (Hoffman and Winder, 2016; Takeshita et al., 2017) are necessary to determine that grip strength progressively decline with increasing age.

### Anxiety-Like Behavior in the Mouse Does Not Mimic Clinical Anxiety

Patients’ self-ratings on the Center for Epidemiologic Studies Depression (CES-D) and trait anxiety are highly correlated (Orme et al., 1986). Thus, we reasoned that performance on the CES-D would be representative for trait anxiety in humans, allowing us to compare it with anxiety-like behavioral assessments in the mouse (Table 2), specifically time for immobile in the open field test. The estimated age-related pattern for immobile time steadily increased with increasing age, while CES-D scores had a U shape, with higher levels of anxiety at young and old ages (Figure 12E). Given that the prevalence of higher level of anxiety in human children and adolescents is found in the educational context (Zeidner, 2014), this high anxiety in early stage of life might be specific phenomenon to human. When we focus on the higher level of anxiety in the latter half of life, therefore, male mice that aged 12 months correspond roughly to humans in their sixties.

It should be noted, however, that results on assessing anxiety-like behaviors in rodents remains controversial. Earlier studies using time spent in the center of the open field show that anxiety level does not change with advancing age (Nolte et al., 2019), and older mice appear to exhibit increased anxiety-like behavior in a light/dark transition test but also show decreased anxiety-like behaviors in the elevated plus maze test (Shoji et al., 2016). For these reasons and a possible confound of simply decreased locomotor activity (see “Physical Aging of Mice Is More Accelerated Than We Had Expected” in the Results), increased neophobia (Crane and Ferrari, 2017) and decreased motivation (Jackson et al., 2021) with aging, a different task such as the elevated plus maze (Pellow et al., 1985), zero maze (Shepherd et al., 1994), and the forced swim test (Porsolt et al., 1977) should be considered for assessing emotional behavior in the aged mouse.

### Two Different Aspects of Memory

Impaired memory is one of the most common cognitive features of non-demented elderly individuals (Craik and Jennings, 1992). Our findings on age-related memory impairment are consistent with previous studies (Rosenzweig and Barnes, 2003; Maei et al., 2009; Kennard and Woodruff-Pak, 2011; Tarantini et al., 2019). Among various cognitive tests to detect memory impairment in the elderly, we selected the Montreal Cognitive Assessment (MoCA) as representative index for human memory (Nasreddine et al., 2005).

For hippocampus-dependent memory performance, we observed two different slopes of pattern dependent on level of attention required (Figures 12F,G). Memory performance that requires a low level of attention showed similar age-related pattern for human and mouse (Figure 12F). In contrast, age-related memory impairment are exaggerated when more attention is required in male mice begins to decline relatively earlier and at a faster rate compared to MoCA performance in humans (Figure 12G). The analogical results in human are found in previous study showing that age-related differences are more pronounced as memory tasks are made more complex by increasing the amount of attention required (McDowd and Craik, 1988). Considering these, age-related memory impairment in elderly humans assessed by MoCA can be detected in male mice at 22 months of age (low level of attention), as well as in male mice at 12 months of age (high level of attention). Concomitantly, our results emphasize that multiple parameters of memory performance must be considered when defining an aging phenotype.

## Conclusion

One of major challenge in the field of aging is to evaluate the effect of an intervention. When we develop the interventions in mouse model of aging, we clearly know that it should be evaluated when their physiological function is sufficiently decreased but has a room for the improvement that provide the opportunity research on the intervention. Despite C57BL/6J mice is widely available and commonly used as animal model of aging research (Mitchell et al., 2015), we have blind acceptance of 24-month-old mice as the golden standard of old age of mice. In the present study, we showed that the pattern of functional aging of the C57BL/6J male mouse are non-linear and unique to each behavioral trait. As chronological age alone is not a reliable indicator of aging for the elderly (Sharkey, 1987), aging of mouse should be judged on the basis of its physiological functions. The results of our study suggest new ways to design aging experiments with male mice that correspond more closely to the phenotype of human aging. Moreover, our results imply that interventions for frailty that affect more than one function may be more beneficial than those that affect just one system.

In the present study, male mice were used in accordance with a tradition in scientific research. Recent evidence, however, reveals the substantial sex-based differences in mice behavior (Jonasson, 2005; Spröwitz et al., 2013; Matsuda et al., 2015; Tucker et al., 2016). Therefore, using a balanced population of male and female mice are advocated (Wald and Wu, 2010; Shansky, 2019). The detailed characterization in behaviors of female mice using behavioral test battery will provide useful information for designing behavioral experiments, interpreting mouse phenotypes, understandingthe neurobiological basis of age-related behavioral changes endowed with potential to translate in the context of clinical settings.

## Data Availability Statement

The raw data supporting the conclusions of this article will be made available by the authors, without undue reservation, to any qualified researcher.

## Ethics Statement

The animal study was reviewed and approved by Animal Experiment Committee of the Tokyo Metropolitan Institute of Gerontology (Animal Protocol Approval Numbers 17012 and 20018).

## Author Contributions

SY and SE designed the study. SY conducted the experiments and data analysis. SY and SE interpreted the data and wrote the manuscript. Both authors contributed to manuscript revision, read, and approved the submitted version.

## Conflict of Interest

The authors declare that the research was conducted in the absence of any commercial or financial relationships that could be construed as a potential conflict of interest.

## Publisher’s Note

All claims expressed in this article are solely those of the authors and do not necessarily represent those of their affiliated organizations, or those of the publisher, the editors and the reviewers. Any product that may be evaluated in this article, or claim that may be made by its manufacturer, is not guaranteed or endorsed by the publisher.
