# Supplementary material for: Functional Aging in Male C57BL/6J Mice Across the Life-Span: A Systematic Behavioral Analysis of Motor, Emotional, and Memory Function to Define an Aging Phenotype
Source: Front Aging Neurosci. 2021 Aug 2;13:697621. doi: 10.3389/fnagi.2021.697621 (PMC8365336; doi:10.3389/fnagi.2021.697621)
Supplement: Supplementary file 1 [file Data_Sheet_1.PDF]

Supplementary materials for " Functional aging in male C57BL/6J mice across the life-span: A systematic behavioral analysis of motor, emotional, and memory function to define an aging phenotype "

by Yanai and Endo

Open field test

Mice in the behavioral test battery group were tested on the open field when they reached 3, 6, 12, 18, or 22 months of age. Briefly, they were placed in the empty field and allowed to explore for 15 min on two successive days. In the open field test, distance traveled and the number of rearing reflect locomotor activity, whereas the time spent in the center and immobility time reflect anxiety level. These behaviors were assessed under relatively dark illumination (10 lx) on the first day, and under bright illumination (300 lx) on the second day. Habituation to the novel environment were assessed by calculating 3 min moving average for each performance measure. Regardless of light condition, all five age cohorts reached asymptote performance within 15 min trial in all parameters.

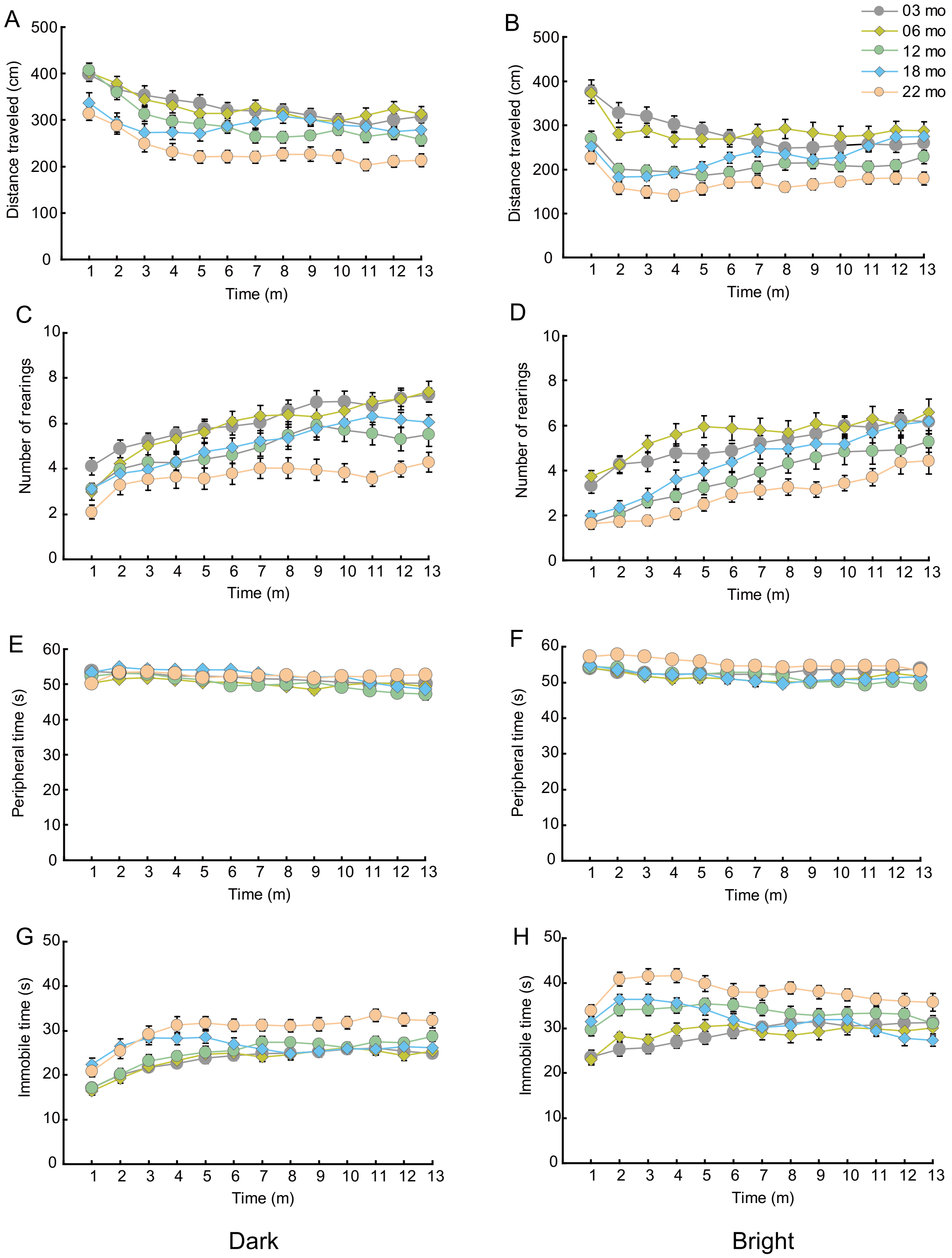

Fig. S1. Open field test. Distance traveled, number of rearings, peripheral time, and immobile time were expressed as 3 min moving average. Mice in all five age cohorts reached asymptote performance withing 15 min trial in all parameters under both light conditions.
