# Supplementary material for: Functional Aging in Male C57BL/6J Mice Across the Life-Span: A Systematic Behavioral Analysis of Motor, Emotional, and Memory Function to Define an Aging Phenotype
Source: Front Aging Neurosci. 2021 Aug 2;13:697621. doi: 10.3389/fnagi.2021.697621 (PMC8365336; doi:10.3389/fnagi.2021.697621)
Supplement: Supplementary file 2 [file Table_1.PDF]

Supplemental Table. Summary of statistical analyses

Analysis / Task	Statistical value
General physical conditions	
Body weight *	F (4, 112) = 57.963, p < 0.001
Food intake *	F (4, 112) = 0.319, p = 0.865
Water intake *	F (4, 112) = 1.666, p = 0.163
Physical activity	
Distance traveled (Open field test) **	
main effect of age	F (4, 112) = 14.348, p < 0.001
main effect of light condition	F (1, 112) = 2202.846, p < 0.001
interaction (age x light condition)	F (4, 112) = 3.924, p = 0.005
Number of rearings (Open field test) **	
main effect of age	F (4, 112) = 10.765, p < 0.001
main effect of light condition	F (1, 112) = 28.711, p < 0.001
interaction (age x light condition)	F (4, 112) = 1.327, p = 0.264
Distance traveled (Home-cage activity) *	F (4, 112) = 14.418, p < 0.001
Gait speed	
Maximum gait speed (Rotarod test) *	F (4, 112) = 21.325, p < 0.001
Grip strength	
Wire hanging test *	F (4, 112) = 21.104, p < 0.001
Anxiety	
Peripheral time (Open field test) **	
main effect of age	F (4, 112) = 3.460, p = 0.01
main effect of light condition	F (1, 112) = 24.203, p < 0.001
interaction (age x light condition)	F (4, 112) = 2.988, p = 0.022
Immobile time (Open field test) **	
main effect of age	F (4, 112) = 10.893, p < 0.001
main effect of light condition	F (1, 112) = 398.069, p < 0.001
interaction (age x light condition)	F (4, 112) = 3.299, p = 0.014
Immobile time (Marble burying test) *	F (4, 112) = 18.502, p < 0.001
Number of marbles burried (Marble burying test) *	F (4, 112) = 17.809, p < 0.001
Memory	
Escape latency (Spatial acquisition test, Barnes hole board task) **	
main effect of age	F (4, 112) = 3.252, p = 0.015
main effect of training block	F (3, 336) = 219.725, p < 0.001
interaction (age x training block)	F (12, 336) = 1.880, p = 0.036
Escape distance (Spatial acquisition test, Barnes hole board task) **	
main effect of age	F (4, 112) = 6.403, p < 0.001
main effect of training block	F (3, 336) = 180.888, p < 0.001
interaction (age x training block)	F (12, 336) = 2.631, p = 0.002
Quadrant time (Probe test, Barnes hole board task) *	F (4, 112) = 4.458, p = 0.002
Number of nosepokes (Probe test, Barnes hole board task) *	F (4, 112) = 11.092, p < 0.001
Escape latency (Spatial acquisition training, Morris water maze task) **	
main effect of age	F (4, 112) = 8.614, p < 0.001
main effect of training block	F (9, 1008) = 68.959, p < 0.001
interaction (age x training block)	F (36, 1008) = 0.922, p = 0.602
Swim distance (Spatial acquisition training, Morris water maze task) **	

main effect of age	F (4, 112) = 4.582, p = 0.002
main effect of training block	F (9, 1008) = 71.682, p < 0.001
interaction (age x training block)	F (36, 1008) = 1.148, p = 0.254
Quadrant time (Probe test, Morris water maze task) *	F (4, 112) = 1.681, p = 0.159
Platform crossing (Probe test, Morris water maze task) *	F (4, 112) = 4.329, p = 0.003
Escape latency (Cued training, Morris water maeze task) **	
main effect of age	F (4, 112) = 4.934, p < 0.001
main effect of training day	F (3, 336) = 137.685, p < 0.001
interaction (age x training day)	F (12, 336) = 0.986, p = 0.462
Escape distance (Cued training, Morris water maeze task) **	
main effect of age	F (4, 112) = 1.768, p = 0.140
main effect of training day	F (3, 336) = 45.203, p < 0.001
interaction (age x training day)	F (12, 336) = 1.223, p = 0.266
Swim speed (Morris water maze task) *	
Spatial acquisition training	F (4, 112) = 6.814, p < 0.001
Probe test	F (4, 112) = 19.514, p < 0.001
Cued training	F (4, 112) = 13.617, p < 0.001
Fear conditioning task *	
Pre-Cue (1 hr)	F (4, 112) = 6.967, p < 0.001
Cue-dependent memory test (1 hr)	F (4, 112) = 3.905, p = 0.005
Pre-Cue (24 hrs)	F (4, 112) = 10.005, p < 0.001
Cue-dependent memory test (24 hrs)	F (4, 112) = 1.514, p = 0.203
Context-dependent memory test (48 hrs)	F (4, 112) = 16.503, p < 0.001
Pain sensation	
Electrical footshock sensitivity test *	
Pawflick	F (4, 112) = 3.309, p = 0.013
Vocalization	F (4, 112) = 2.820, p = 0.028
Hotplate test *	F (4, 112) = 0.809, p = 0.522
Spatial memory retention	
Escape latency (Spatial acquisition training, Morris water maze task) **	
main effect of age	F (4, 205) = 15.431, p < 0.001
main effect of training block	F (9, 1845) = 121.269, p < 0.001
interaction (age x training block)	F (36, 1845) = 1.107, p = 0.305
Swim distance (Spatial acquisition training, Morris water maze task) **	
main effect of age	F (4, 205) = 6.743, p < 0.001
main effect of training block	F (9, 1845) = 131.239, p < 0.001
interaction (age x training block)	F (36, 1845) = 1.111, p = 0.300
Platform crossing (Probe test, Morris water maze task) **	
main effect of age	F (4, 195) = 4.324, p = 0.002
main effect of retention days	F (2, 195) = 6.895, p < 0.001
interaction (age x retention days)	F (8, 195) = 0.941, p = 0.484
Quadrant time (Probe test, Morris water maze task) **	
main effect of age	F (4, 195) = 0.741, p = 0.565
main effect of retention days	F (2, 195) = 13.188, p < 0.001
interaction (age x retention days)	F (8, 195) = 1.198, p = 0.302

* One-way ANOVA
** Two-way ANOVA
